# Evaluating the efficacy of cannabidiol to manage surgically induced neuropathic pain in a preclinical rat model: Are T cells a sexually dimorphic target?

**DOI:** 10.1080/24740527.2019.1612235

**Published:** 2019-07-30

**Authors:** K. Linher-Melville, G. Singh

**Affiliations:** a Michael G. DeGroote Institute for Pain Research and Care, McMaster University, Hamilton, Ontario, Canada; b Department of Pathology and Molecular Medicine, McMaster University, Hamilton, Ontario, Canada

**Keywords:** neuropathic pain, chronic pain, sex differences, cannabinoids, immune system, behavior, electrophysiology, nociception

## Abstract

**Background**: Considering the poorly understood etiology and complex symptoms of chronic neuropathic pain (NP), the lack of effective treatments, and sex-dependent differences in the neuroimmune system as well as in antinociceptive responses to existing pharmacological agents, the potential to therapeutically target the endocannabinoid system as a means of treating this type of intractable pain is clinically relevant and timely. Chronic NP may involve the utilization of distinct immune cell populations in males and females that differentially affect supraspinal and spinal neuromodulation. It is therefore important to investigate the effects of cannabidiol (CBD) on chronic NP-induced nociceptive responses in both sexes.

**Aims**: Evaluating whether the expression of markers associated with CD4^+^ T cells are affected by CBD in a sexually dimorphic manner will provide key insights into the contribution of these adaptive immune cells to the onset and progression of NP.

**Methods**: Future research will be directed toward examining the potential sex-dependent effects of this nonpsychotropic cannabinoid relative to vehicle in a preclinical model of chronic postsurgical NP. Specifically, (1) differences in nociceptive behavior, (2) chronic changes in neural firing patterns, and (3) up- or downregulation of markers associated with CD4^+^ T cells in relevant tissues will be evaluated to better understand CBD-mediated neuroimmune modulatory effects in males and females.

**Conclusions**: Chronic postsurgical pain is a growing clinical problem. Current treatment strategies rely on opioid-based therapeutics, which affect patient quality of life and are associated with addiction and withdrawal. Treatment of nerve injuries with CBD could provide an effective alternative to manage NP. Understanding its mechanisms of action will provide important insights into the sex-dependent application of this nonpsychoactive cannabinoid, setting the groundwork for large-scale Canadian clinical trials in women and men presenting with chronic pain.

## RÉSUMÉ

**Contexte**: Compte tenu de la mauvaise compréhension de l’étiologie et des symptômes complexes de la douleur neuropathique chronique, de l’absence de traitements efficaces et des différences entre les sexes dans le système neuro-immunitaire ainsi que dans les réponses antinociceptives aux agents pharmacologiques existants, la possibilité que le système endocannabinoïde soit la cible thérapeutique pour traiter ce type de douleur irréductible est opportun et pertinent sur le plan clinique. La neuropathie chronique peut impliquer des populations de cellules immunitaires distinctes chez les mâles et les femelles affectant la neuromodulation supraspinale et spinale de manière différenciée. Il est donc important d’investiguer les effets du cannabidiol (CBD) sur les réponses nociceptives induites par la douleur neuropathique chronique chez les deux sexes.

**But**: Le fait de déterminer si l’expression des marqueurs associés aux cellules CD4 + T est affectée par le CBD d’une manière sexuellement dimorphique permettra de mieux comprendre la contribution de ces cellules immunes adaptatives au déclenchement et à la progression de la douleur neuropathique.

**Méthodes**: Les prochaines études porteront sur l’examen des effets différenciés selon le sexe de ce cannabinoïde non psychotrope dans un modèle préclinique de douleur neuropathique post-chirurgicale chronique. En particulier, 1) les différences dans le comportement nociceptif, 2) les changements chroniques dans les modes de décharge neurale, et 3) la régulation à la hausse ou à la baisse des marqueurs associés aux cellules CD4+ T dans les tissus pertinents seront évalués pour mieux comprendre les effets neuro-immunitaires occasionnés par le CBD chez les mâles et chez les femelles.

**Conclusions**: La douleur post-chirurgicale chronique est un problème clinique croissant. Les stratégies de traitement actuelles s’appuient sur des substances thérapeutiques à base d’opioïdes qui affectent la qualité de vie du patient et qui sont associées à la dépendance ainsi qu’à l’état de manque. Le traitement de blessures aux nerfs à l’aide du CBD pourrait être une option de rechange efficace pour la prise en charge de la douleur neuropathique. La compréhension de ses mécanismes d’action donnera des indications importantes quant à l’utilisation différenciée selon le sexe de ce cannabinoïde non psychoactif, préparant ainsi le terrain pour des essais cliniques canadiens de grande envergure auprès de femmes et d’hommes souffrant de douleur chronique.

## Background

### Neuropathic pain

Damage to nerves of the peripheral nervous system (PNS) may result in the development of chronic intractable pain referred to as neuropathic pain (NP).^1^ The rising prevalence of NP, which is of diverse etiology and may be induced by infection, diabetes, cancer, neurotoxic or traumatic injury, and surgery, makes it a major clinically and socially relevant issue.^2^ Patients with NP experience spontaneously generated ongoing or paroxysmal pain or induced pain, including static or dynamic mechanical and thermal allodynia/hyperalgesia.^3,4^ This pain is a result of pathological changes in the PNS characterized by aberrant neurotransmission, as well as changes in signal processing mechanisms in the central nervous system (CNS). In response to an insult that contributes to NP, reduced thresholds to action potential firing, neuronal death and degradation, irregular neuronal sprouting, inflammation of neuronal and support cells (astrocytes, microglia), and demyelination of peripheral nerves may occur.^5^ In a state of NP, nociceptors undergo pathological changes that include aberrant neuronal discharge.^5,6^ The resulting changes in the PNS and CNS have negative effects on patient health and well-being, including disability and treatment-induced side effects, which become intractable over time. Preventing the onset of this pathological pain state following nervous tissue damage and improving patient quality of life are primary clinical concerns that require the development and implementation of more effective therapeutic options.

### Cannabinoids

For most patients, current pharmacological NP treatment options provide limited analgesic relief and are often accompanied by adverse side effects.^7^ Alternatives, including cannabinoids (CBs), are beginning to be systematically explored to more effectively treat NP. The primary function of the endocannabinoid (eCB) system, which is highly conserved across species, is to maintain physiological homeostasis.^8^ eCB neuromodulation contributes to normal endocrine function, cognition/memory, immune recognition, inflammation, and antinociceptive responses.^8,9^ The equilibrating effects on stress and pain suggest that targeting this system may be therapeutically promising, especially given its efficacy in managing pain associated with fibromyalgia.^10^ CB receptors (CBRs) and their various ligands are distributed throughout the PNS and CNS, as well as in other peripheral tissues, including the immune system.^11^ The function of eCBs identified to date that interact with CBRs in the nervous system (primarily CBR1) and the periphery (primarily CBR2) have only been partially elucidated, but preclinical studies support a role in modulating nociception.^10,11^

Of the more than 450 compounds present in *Cannabis sativa*, two CBs are currently of particular clinical relevance for treating NP, including delta 9-tetrahydrocannabinol (Δ9-THC), which has psychoactive and analgesic properties, and CBD, which does not affect cognition, memory, or mood but elicits antinociceptive effects. Findings from clinical trials with *Cannabis sativa*–based medicinal extracts as well as synthetic CBs suggest that targeting the eCB system may be a promising approach to managing chronic NP.^9,12^ In addition to modulating nociceptive signals, CBs, especially CBD, may also counter inflammatory responses contributing to chronic NP.^13^

### Sexual dimorphisms in immune responses to pain

Psychosocial aspects such as coping strategies and tolerance contribute to differences in the prevalence and reporting of chronic pain in men and women,^14–16^ and biological factors related to immune system function (reviewed in Sorge and Totsch^17^), brain structure and activity,^18,19^ and analgesic responses to drugs^20,21^ may also contribute to differences in pain responses in males and females. Nociceptive behaviors such as mechanical hyperalgesia are mediated primarily by spinal microglia in male mice, whereas in female mice, the response preferentially involves T cells.^17,22^ Male reliance on microglia appears to be dependent on testosterone,^22^ which plays a role in suppressing T cell–mediated responses (reviewed in Sorge and Totsch^17^ and Trigunaite et al.^23^).

Adaptive immunity involves responses to specific stimuli that initiate CD4^+^ T cell development in the thymus. The activation of distinct signaling pathways then induces their differentiation into specialized subsets of T lymphocytes, including T helper (Th) 1, Th2, Th9, Th17, Th22, Tmog (T cells that are specific for the autoantigen, myelin oligodendrocyte glycoprotein (MOG), found in multiple sclerosis), and Treg (regulatory T cells) cells, which then undergo expansion in the spleen. Activated CD4^+^ T cell subsets secrete defined combinations of cytokines, enabling them to recruit/activate other immune cells, dampen immune responses, or maintain immune memory. Adaptive immunity differs between men and women (reviewed in Giefing-Kroll et al.^24^). The ratio of Th1 cells (primarily pro-inflammatory, driving cell-mediated immunity) relative to Th2 cells (primarily anti-inflammatory, mediating humoral immune responses) may be sexually dimorphic.^25^ Indeed, testosterone and its metabolites suppress the differentiation of, and interferon (IFN)-γ secretion by, splenic Th1 cells, shifting the distribution toward the Th2 subtype.^26^ Furthermore, men with androgen deficiencies have higher levels of the pro-inflammatory cytokines interleukin (IL)-2, IL-1β, and tumor necrosis factor-α, as well as altered T cell ratios.^27^ Immune recruitment of Th1 or Th2 subsets is regulated by specific cytokines. IL-12 induces development of the Th1 subset, enhancing their production/secretion of IFN-γ and IL-2 to promote subsequent humoral immune responses.^28–32^ In contrast, IL-4 decreases the production of Th1 cells while inducing the development of Th2 cells, which, in turn, secrete IL-4.^28,29,33,34^ Importantly, CBD suppresses murine T cell function, downregulating the production/secretion of IFN-γ and IL-2 from purified splenic T cells,^35^ and it will therefore be of interest to mechanistically examine its neuroimmune modulatory effects during the onset and progression of chronic NP in males and females.

## Future directions

### Effect of CBD on nociceptive behavior

Male and female Sprague Dawley rats will be implanted with a sciatic nerve cuff, which is known to induce significant nociceptive behavior.^36^ On the day of surgery, once-daily oral gavage with CBD oil or medium chain fatty acids (vehicle) will commence, continuing for 14 consecutive days. Weekly Von Frey paw withdrawal testing will be carried out until 9 weeks postsurgery to monitor changes in nociceptive behavior. This set of experiments will demonstrate whether CBD is effective at blocking evoked allodynia/hyperalgesia and spontaneous pain behavior in a sexually dimorphic manner, providing important insights into the clinical relevance of this cannabinoid for managing postsurgical pain in men and women.

### Effect of CBD treatment on neural firing patterns

Animals that exhibit significant changes in nociceptive behavior in response to CBD treatment will be subjected to endpoint electrophysiological recordings via soma stimulation at relevant DRG associated with the lumbar 4 to 6 spinal cord region, which integrates peripheral signals via the sciatic nerve, as routinely carried out by our group.^37^ Electrophysiological recordings will facilitate dissemination of which specific nerve fibers are being impacted by early postsurgical CBD treatment. Though CBD may mediate its antinociceptive effects by suppressing T cell activity,^35^ it has also been shown to act as a direct agonist/desensitizer of recombinant rat transient receptor potential vanilloid 1 (TRPV1), TRP channels of subfamily V type 2 (TRPV2), and subfamily A type 1 (TRPA1) in an in vitro model of epileptiform activity, demonstrating potential to treat neuronal hyperexcitability.^38^

### Effect of CBD on markers associated with CD4^+^ T cells

Relevant tissues will be collected from each animal at the experimental endpoint. The presence of Th1 and Th2 cells will be distinguished at the mRNA level using markers that differentiate between these two subsets, with Th1 cells expressing IL-2 receptor alpha and beta chains,^30^ whereas only Th2 cells express a fully functional IL-1 receptor.^31^ These data will be correlated with behavioral nociceptive profiles and electrophysiological recordings. Given that males and females display neuroimmune differences and CBD may modulate inflammatory and immune responses, assessing changes in Th1 and Th2 cell–associated markers may provide important insights into sex differences that may arise in response to treatment with this cannabinoid.

## Conclusions

Chronic pain is a growing clinical problem, and current treatment strategies primarily rely on opioid-based therapeutics, which adversely affect patient quality of life and are associated with potential addiction and/or withdrawal.^39^ Administration of neuroprotective agents, including CBD, could emerge as an effective method to treat, or potentially even prevent, postsurgical NP. It is important to understand sexual dimorphisms in overall treatment efficacy and how the neuroimmune system, particularly with respect to the currently understudied T cell component, may play different roles in women and in men to modulate therapeutic outcomes.
